# Long‐term efficacy of no‐touch radiofrequency ablation in the treatment of single small hepatocellular carcinoma: A single center long‐term follow‐up study

**DOI:** 10.1002/cam4.5428

**Published:** 2022-11-29

**Authors:** Guodong Wu, Jing Li, Changfeng Li, Xia Ou, Kai Feng, Feng Xia, Zhiyu Chen, Leida Zhang, Kuansheng Ma

**Affiliations:** ^1^ Institute of Hepatobiliary Surgery Southwest Hospital, Army Medical University Chongqing China; ^2^ Department of Hepatobiliary Surgery The 958th Hospital of the PLA Army Chongqing China

**Keywords:** hepatocellular carcinoma, local tumor progression, no‐touch, radiofrequency ablation, survival rate

## Abstract

**Objective:**

To evaluate the long‐term efficacy of no‐touch radiofrequency ablation (NT‐RFA) for treating single hepatocellular carcinoma (HCC) less than 3 cm.

**Methods:**

A total of 331 patients with HCC less than 3 cm undergoing RFA in Southwest Hospital from 2015 to 2020 were analyzed retrospectively. All patients were divided into NT‐RFA group (*n* = 113) and conventional RFA (C‐RFA) group (*n* = 218). The survival rate, local tumor progression (LTP) and intrahepatic distant recurrence (IDR) of the two groups were calculated and compared.

**Results:**

A significant difference was observed in ablation range (*p* = 0.000) and safety margin (*p* = 0.000) between the two groups. The 1‐, 2‐, 3‐, 4‐and 5‐year overall survival (OS) rates in NT‐RFA and C‐RFA group were 99.12%, 93.73%, 76.18%, 57.00%, 45.17% and 99.08%, 89.91%, 71.26%, 54.28%, 41.77%, respectively. There was no significant difference between the two groups (*p* = 0.281). The 1‐, 2‐, 3‐, 4‐and 5‐year recurrence‐free survival (RFS) rates in NT‐RFA and C‐RFA group were 78.51%, 52.59%, 41.02%, 34.36%, 30.92% and 68.81%, 44.95%, 30.88%, 23.73%, 22.88%, respectively. The two groups differed significantly (*p* = 0.044). The 1‐, 3‐and 5‐year LTP‐free survival rates in NT‐RFA and C‐RFA group were 87.12%, 74.99%, 72.32% and 75.75%, 65.52%, 65.52%, respectively. The two groups also differed significantly (*p* = 0.024). Furthermore, the RFS rates of D ≤ 2 cm subgroups in NT‐RFA and C‐RFA groups differed significantly (*p* = 0.037), while the RFS rates of 2 cm < D ≤ 3 cm subgroups in two groups showed no significant difference (*p* = 0.578).

**Conclusions:**

The RFS rates of single HCC less than 3 cm treated by NT‐RFA was significantly higher than that of C‐RFA. Due to a larger ablation range and safety margin, NT‐RFA could significantly reduce LTP and improve RFS. Dual‐electrode NT‐RFA can significantly improve the RFS rate of patients with HCC less than 2 cm, but there is no obvious advantage compared with C‐RFA in the treatment of HCC over 2 cm.

## Graphical abstract

This study is the first comprehensive summary of the experience of using no‐touch radiofrequency(NT‐RFA) to treat patients with single HCC less than 3cm in a single center in the past 6 years. All RFA operations were implemented according to unified procedures, with the largest number of cases and the longest duration of follow‐up. In particular, the advantages and disadvantages of NT‐RFA technology are comprehensively analyzed, and the range of HCC patients who can benefit from NT‐RFA is proposed for the first time.

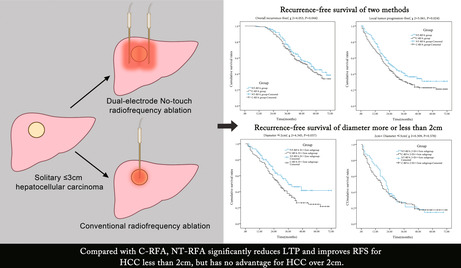

## 1 INTRODUCTION

Radiofrequency ablation (RFA) has been regarded as one of the definitive treatments for hepatocellular carcinoma (HCC), especially for early stage HCC.^1^, ^2^ It has been proved that RFA has no significant difference in the long‐term efficacy from other treatments for early stage HCC, such as surgical resection, microwave ablation and cryoablation.^3^, ^4^, ^5^, ^6^ However, conventional RFA treatment requires insertion of the electrode into the tumor, which is not in line with the tumor free principle of tumor surgical treatment. At the end of the 19th century (1890), Halsted put forward the tumor free principle of tumor surgical treatment. This principle has been recognized and valued by doctors all over the world, and has been continuously developed and improved. It has been applied to various fields of tumor surgery, especially in the surgical treatment of HCC. Therefore, no‐touch RFA (NT‐RFA) emerged and has become increasingly popularized. In 2014, Olivier et al. were the first to report a clinical study of applying NT‐RFA to the treatment of early stage HCC.^7^ In fact, NT‐RFA technology has been used in clinical practice for more than 10 years, and numerous studies have found that NT‐RFA technology can improve the therapeutic effect of HCC.^8^, ^9^, ^10^ However, long‐term observation and systematic study of NT‐RFA efficacy remain scarce, no unified standards are available for application of this technology to the treatment of HCC, and controversies exist over the scope of its application. Since 2015, we have been using dual‐electrode NT‐RFA to treat early stage HCC in our center, and collected a large number of cases and gained experience. Through a long‐term follow‐up of 331 patients with HCC, this study analyzed and compared the long‐term efficacy between dual‐electrode NT‐RFA and conventional single electrode RFA in the treatment of single HCC less than 3 cm, to assess the value of dual‐electrode NT‐RFA in HCC treatment.

## 2 PATIENTS AND METHODS

### 2.1 Basic information of patients

This study included single HCC patients treated with C‐RFA or NT‐RFA in our center from December 2015 to December 2020. A total of 353 cases were obtained, among which 22 (6.23%) were lost to follow up, and a total of 331 cases were finally included in this study. There were 277 male and 54 female patients (male to female ratio 5.1:1). NT‐RFA group had 113 patients (96 males and 17 females), with a mean age of 53.06 ± 10.82 (33–82) years, while C‐RFA group had 218 patients (181 males and 37 females), with a mean age of 54.16 ± 9.97 (28–77) years. All patients had no contraindications to RFA and signed an informed consent. The advantages and disadvantages of C‐RFA and NT‐RFA treatments were informed to the patients, and the patients chose one of them voluntarily. This study was approved by the Research Ethics Committee of the Southwest Hospital, the First Affiliated Hospital of Army Medical University.

### 2.2 Diagnosis of HCC

History taking and physical examination were performed after admission, and necessary auxiliary examinations and evaluation were completed before operation. All patients were diagnosed with HCC according to the Guidelines for the Diagnosis and Treatment of Hepatocellular Carcinoma (2019 Edition).^2^ Necessary laboratory tests include hepatitis virus markers and AFP, and imaging examinations included contrast‐enhanced ultrasound (US), dynamic contrast‐enhanced computed tomography (CT) and multimodal magnetic resonance imaging (MRI) scans. Clinical diagnosis of HCC was based on the history of chronic liver disease, level of AFP, and the characteristic “wash in and wash out” enhancement pattern revealed by imaging examinations. All patients in NT‐RFA group and 155 patients in C‐RFA group were diagnosed by imaging and laboratory examination, and 63 patients in C‐RFA group were diagnosed by percutaneous liver biopsy.

### 2.3 Inclusion and exclusion criteria

Inclusion criteria were patients with clinical or pathological diagnosis of HCC with a single lesion and diameter ≤3 cm, Child‐Pugh grade A or B liver function. Exclusion criteria were other definitive treatment or liver transplantation before RFA, extrahepatic metastasis and combined with other neoplastic diseases, Child‐Pugh grade C, severe coagulopathy, refractory ascites, hepatic encephalopathy, and the patients who lost follow‐up.

### 2.4 Basic clinical information

Among all cases, 301 (90.94%) had a history of hepatitis (297 of hepatitis B and 4 of hepatitis C), but there was no significant difference between the two groups (*χ* ^2^ = 2.933, *p* = 0.087). There were 143 cases of subcapsular tumors (54 cases in NT‐RFA group and 89 cases in C‐RFA group), and 41 cases of paravascular tumors (16 cases in NT‐RFA group and 25 cases in C‐RFA group). There was no significant difference in tumor location between the two groups. There was also no significant difference in other preoperative examination indexes between the two groups (Table 1). All patient data were collected from the clinical research center of the Institute of Hepatobiliary Surgery of Southwest Hospital, and the accuracy of data was checked.

**TABLE 1:** Clinical data of two groups

Category	NT‐RFA group (*n* = 113)	C‐RFA group (*n* = 218)	*χ* ^2^/*t* value	*p* Value
Sex (M/F)	96/17	181/37	0.203	0.653
Age (years)	53.06 ± 10.82 (33–82)	54.16 ± 9.97 (28–77)	0.961	0.337
Viral infection (no/HBV or HCV)	6/107	24/194	2.933	0.087
Child–Pugh grade (A/B)	105/8	204/14	0.052	0.820
Tumor size (mm)	19.13 ± 5.22(8–30)	18.77 ± 5.56(7–30)	0.617	0.537
Subcapsular tumor (no/yes)	59/54	129/89	1.470	0.225
Perivascular tumor (no/yes)	97/16	193/25	0.497	0.481
Cirrhosis (no/yes)	19/94	39/179	0.060	0.807
Comorbid diseases (no/yes)	78/35	148/70	0.044	0.833
ALT (U/L)	38.79 ± 31.06 (11.1–191.4)	41.48 ± 35.10 (8.8–295.4)	0.710	0.478
AST (U/L)	40.09 ± 28.43 (16.3–242.2)	47.24 ± 46.93 (11.8–462.4)	1.525	0.128
ALP (U/L)	104.32 ± 40.02 (46–396)	106.29 ± 44.37 (39–455)	0.411	0.682
γ‐GT (U/L)	88.02 ± 86.63 (9–665)	89.81 ± 100.51 (6–892)	0.509	0.611
Serum albumin (g/L)	41.79 ± 4.56 (28.5–50.8)	40.81 ± 6.03(26.7–54.5)	1.571	0.117
Total bilirubin (umol/L)	19.86 ± 11.42 (3.44–96.3)	20.25 ± 10.72(6.15–88.0)	0.319	0.750
Total bile acid (umol/L)	12.84 ± 17.32 (0.5–123)	15.89 ± 22.80 (0.3–218.3)	1.289	0.198
Platelet (×10^9^/L)	121.18 ± 55.72 (34–325)	116.62 ± 54.52(31–284)	0.741	0.459
Urea nitrogen (mmol/L)	5.49 ± 1.54 (2.50–11.10)	5.54 ± 1.58(2.03–10.14)	0.304	0.761
Serum creatinine (umol/L)	72.27 ± 15.04 (38.3–115.2)	71.30 ± 18.34(28.7–224.1)	0.501	0.617
PT (s)	12.84 ± 1.54 (8.5–18.0)	13.01 ± 1.62(9.3–18.8)	0.947	0.344
AFP (ng/ml)	182.59 ± 327.35 (0.97–2287)	237.53 ± 813.48(1.1–7500)	0.700	0.484
PIVKA‐II (ng/ml)	95.67 ± 177.88 (9–1145)	132.37 ± 330.59 (1–2725)	0.887	0.376
HBV‐DNA (positive/negative)^a^	24/89	57/161	0.970	0.325

Abbreviations: AFP, alpha‐fetoprotein; ALP, alkaline phosphatase; ALT, alanine aminotransferase; AST, aspartate aminotransferase; γ‐GT, gamma glutamyl transpeptidase; HBV, hepatitis B virus; HBV‐DNA, DNA of hepatitis B virus; HCV, hepatitis C virus; PT, Prothrombin time; PIVKA‐II, protein induced by vitamin K absence or antagonist‐II.

^a^ HBV‐DNA > 1000 cps/ml was considered positive. Independent sample *t* test for continuous variables and Pearson chi‐square test for discrete variables.

### 2.5 Procedure of C‐RFA and NT‐RFA

A disposable biopsy needle (14G × 16 cm, Bard Peripheral Vascular, Inc.) was used for percutaneous liver biopsy before C‐RFA. LDRF‐120 S RFA device (Lead Electron Corporation) and Elektrotom 106 HiTT minimally invasive surgical system (Berchtold Medizin Electronik GmbH) are used for C‐RFA. NT‐RFA was performed using the LDRF‐120 S RFA device (Lead Electron Corporation). All patients were treated under monitoring anesthesia. RFA were performed by two Senior doctors in the center and under the guidance of EUB‐405 ultrasonic diagnostic instrument (Hitachi). C‐RFA uses a single‐needle electrode and the electrode was inserted along the center of the lesion. The initial power is 50 W, then increase 10 W every 2 minutes until reaching the maximum power of 95 W, and maintain this power until the impedance reached the maximum value. The procedure can be repeated until the lesion is covered by the necrotic area. NT‐RFA is to insert two single‐needle electrodes along the tumor‐free area on both sides of the lesion under the guidance of ultrasound. The distance between the two electrodes can be adjusted according to the size of the tumor. The perfusion electrode (Lead Electron Corporation) is used when the distance between the two electrodes exceeds 2.2 cm, and normal saline is slowly injected through the perfusion electrode during ablation. The ablation time and power are the same as that of C‐RFA. Needle path ablation was performed when the electrode was withdrawn. Vital signs were monitored during treatment. Observe for 30 minutes after treatment in the procedure room, and then contrast‐enhanced US was performed again to observe whether there are residual lesions. If residual lesions are found, repeat RFA procedure.

### 2.6 Postoperative efficacy evaluation

Postoperative complications were classified according to Clavien‐Dindo classification standard.^11^ AFP, liver function and blood routine examination were rechecked the next day after RFA procedure. Contrast‐enhanced CT was performed within 3 days after RFA procedure to determine whether there were residual lesions. If residual lesions were found, RFA procedure would be repeated within 1 week. The size of ablation range (the maximum cross‐sectional area) and the safety margin (the minimum distance of the ablation area beyond the boundary of the original lesion) were measured by imaging examination.

### 2.7 Follow‐up and recurrence

One month after RFA, the treatment efficacy was evaluated in each patient. After that, the patients were followed up every 2 months for 1 year, and every 3 months after 1 year. Liver function, AFP, HBV‐DNA and imaging examination (contrast‐enhanced US, dynamic contrast‐enhanced CT or MRI) were performed at each follow‐up. The information of tumor recurrence, treatment after recurrence and cause of death were collected. Recurrence included local tumor progression (LTP), intrahepatic distant recurrence (IDR) and extrahepatic metastasis. LTP was defined as recurrence contiguous to the edge of the original ablation zone and ablation tract, and IDR defined as intrahepatic recurrence away from the original ablation zone. The recurrence free survival rate, overall survival rate, LTP‐free and IDR‐free survival rate were counted. The patients were followed up until death. The duration of the follow‐up ranged 12–72 months, with a mean time of 35.77 ± 16.23 months.

### 2.8 Statistical analysis

The statistical software SPSS 19.0 was used for all statistical analyses. Variables are presented as mean ± standard deviation or number of cases. Continuous variables were compared by *t*‐test, and discontinuous variables by Pearson chi‐square test or Fisher's exact test. Kaplan Meier survival analysis was used to assess the OS rate, RFS rate, LTP‐free and IDR‐free survival rate. Log rank test was used to compare the survival rate. Cox proportional hazard model was used for multivariate analyses. *p* Values less than 0.05 were considered statistically significant.

## 3 RESULTS

### 3.1 Short‐term efficacy

RFA was successful in all patients, and all lesions were completely ablated. There were no deaths during hospitalization. There was no significant difference in operation time, number of times and hospital stay between NT‐RFA group and C‐RFA group. However, there were significant differences in the ablation range between the two groups (*p* = 0.000), and the safety margin of two groups were also significantly different (*p* = 0.000, Table 2). There were 96 cases (84.96%) in NT‐RFA group and 181 cases (83.03%) in C‐RFA group of Clavien grade I, 16 cases (14.16%) in NT‐RFA group and 34 cases (15.60%) in C‐RFA group of Clavien grade II, 1 case (0.88%) in NT‐RFA group and three cases (1.38%) in C‐RFA group of Clavien grade III, and no Clavien grade IV/V cases in either group. There was no significant difference in postoperative complications between the two groups (*p* = 0.868, Table 2).

**TABLE 2:** Short‐term efficacy of two groups

Category	NT‐RFA group (*n* = 113)	C‐RFA group (*n* = 218)	*χ* ^2^/*t* value	*p* Value
Operation time (min)	6.64 ± 3.67 (2–26)	6.35 ± 3.13 (2–20)	0.787	0.432
Number of ablation (1/2 or above)	111/2	211/7	0.584	0.445
Ablation range (cm^2^)	15.25 ± 4.82 (3.99–33.00)	10.68 ± 4.77 (1.30–29.12)	8.736	0.000^a^
Safety margin (mm)	8.86 ± 3.48 (2.00–17.00)	6.32 ± 2.91 (1.00–15.50)	7.314	0.000^a^
Clavien grade (I/II/III)	96/16/1	181/34/3	0.283	0.868
Hospital stay (days)	8.96 ± 4.53 (3–27)	9.51 ± 5.04 (3–49)	1.019	0.309

^a^ There were significant differences in ablation range and Safety margin between the two groups. Independent sample *t* test for continuous variables and Pearson chi‐square test or Fisher's exact test for discrete variables.

### 3.2 Survival outcomes

**FIGURE 1:**
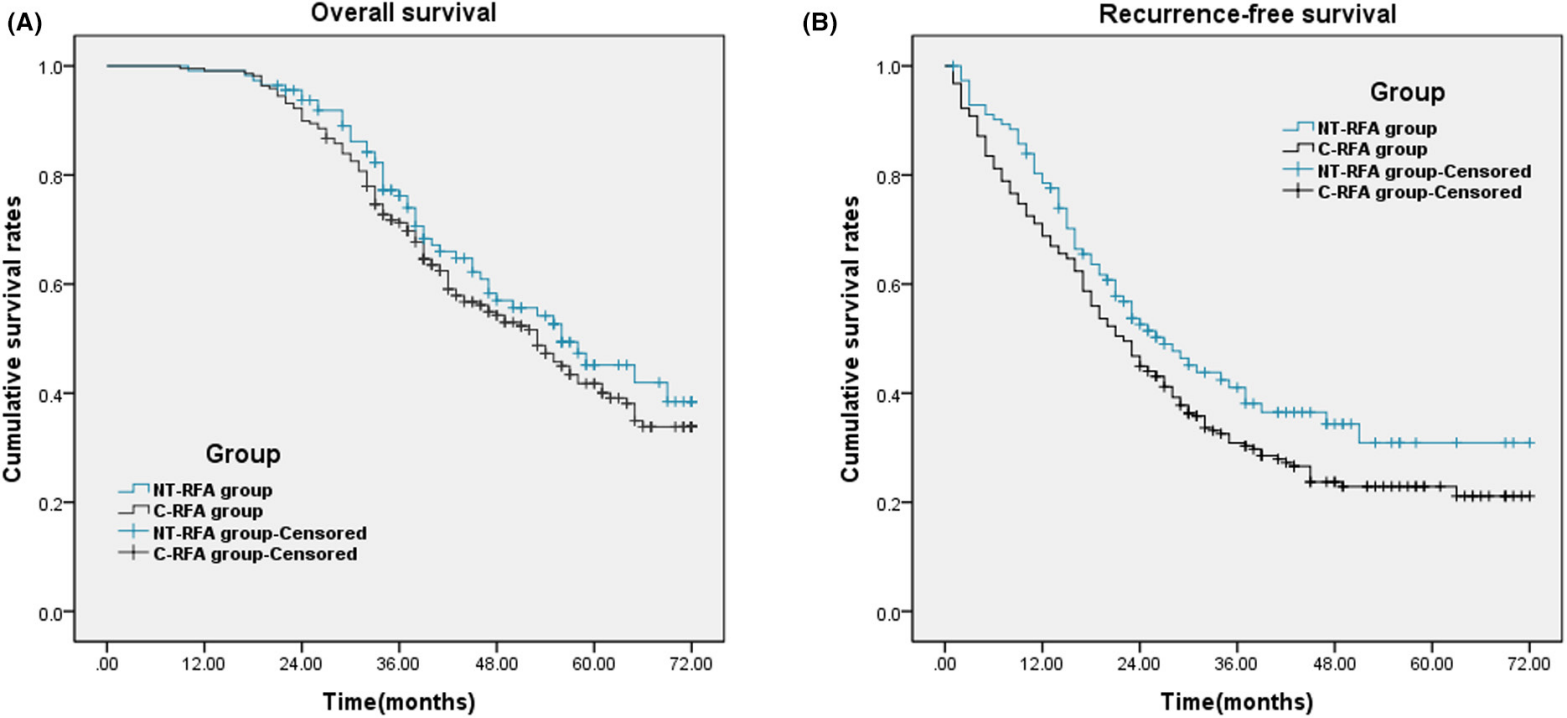
OS and RFS in NT‐RFA and C‐RFA groups. (A) Cumulative OS rates in NT‐RFA group (*n* = 113) and C‐RFA group (*n* = 218). There was no significant difference between the two groups(χ^2^ = 1.161, *p* = 0.281, log‐rank test). (B) Cumulative RFS rates in the two groups. There was a significant difference between the two groups(*χ* ^2^ = 4.053, *p* = 0.044, log‐rank test).

The OS of NT‐RFA group ranged from 10 to 72 months, with a median OS of 56 months. The 1‐, 2‐, 3‐, 4‐ and 5‐ year OS rate was 99.12%, 93.73%, 76.18%, 57.00%, and 45.17%, respectively. The OS of C‐RFA group ranged from 9 to 72 months, with a median OS of 53 months. The 1‐, 2‐, 3‐, 4‐ and 5‐ year OS rate was 99.08%, 89.91%, 71.26%, 54.28%, and 41.77%, respectively. There was no significant difference in OS rate between the two groups (*χ* ^2^ = 1.161, *p* = 0.281, Figure 1A). The RFS of NT‐RFA group ranged from 2 to 72 months, with a median of 26 months. The 1‐, 2‐, 3‐, 4‐ and 5‐ year RFS rate was 78.51%, 52.59%, 41.02%, 34.36%, and 30.92%, respectively. The RFS of C‐RFA group ranged from 1 to 72 months, with a median of 22 months. The 1‐, 2‐, 3‐, 4‐ and 5‐ year RFS rate was 68.81%, 44.95%, 30.88%, 23.73%, and 22.88%, respectively. The RFS rate of NT‐RFA group was significantly higher than that of C‐RFA group (*χ* ^2^ = 4.053, *p* = 0.044, Figure 1B).

### 3.3 HCC recurrence

**FIGURE 2:**
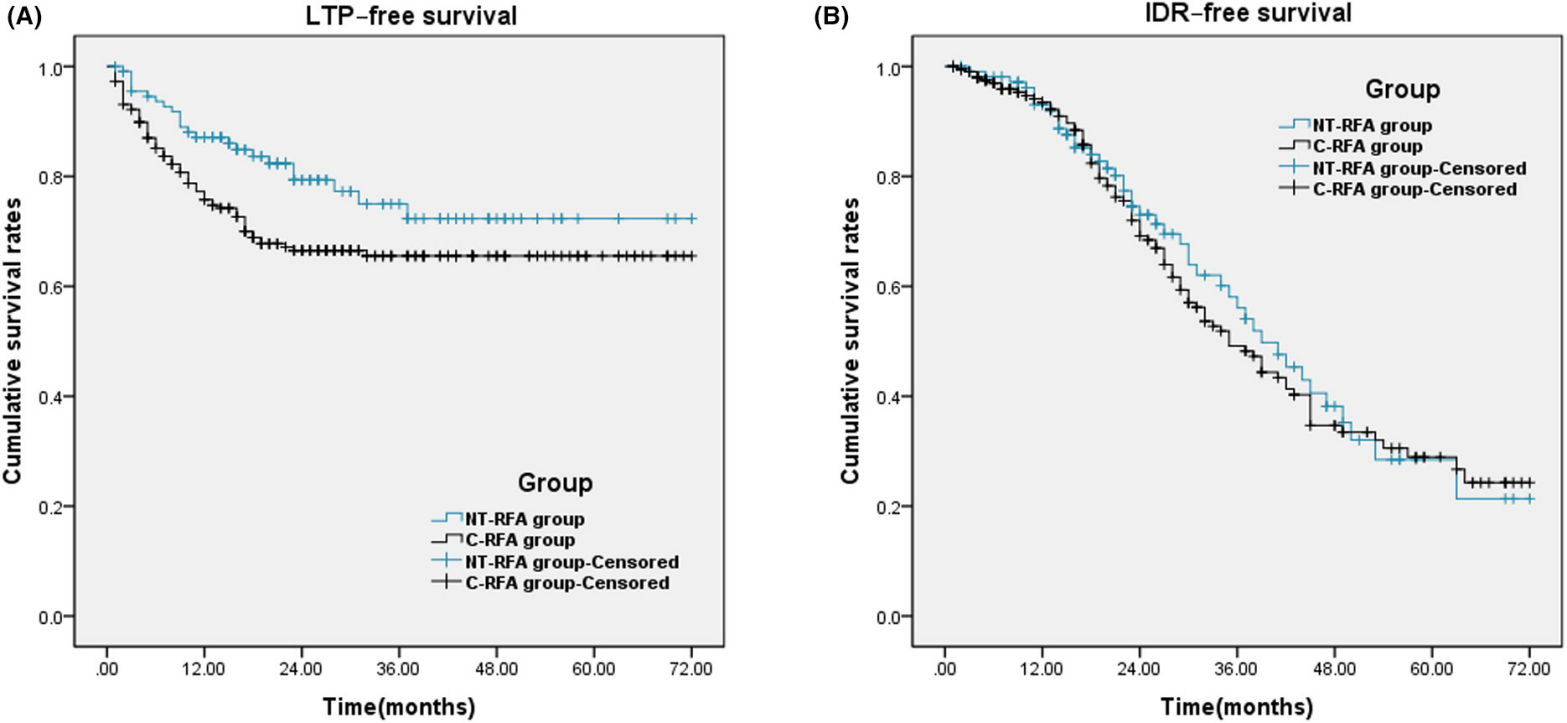
LTP–free survival and IDR‐free survival in NT‐RFA and C‐RFA groups. (A) Cumulative LTP–free survival rates in NT‐RFA and C‐RFA groups. There was a significant difference between the two groups (χ^2^ = 5.061, *p* = 0.024, log‐rank test). (B) Cumulative IDR–free survival rates in the two groups. There was no significant difference between the two groups (*χ* ^2^ = 0.185, *p* = 0.667, log‐rank test).

In this study, 229 patients (69.18%) were diagnosed with HCC recurrence or metastasis during the follow‐up period, including 67 (59.29%) cases in NT‐RFA group and 162 (74.31%) in C‐RFA group. There was a significant difference between the two groups. Among the patients with HCC recurrence, 92 were LTP, including 23 in NT‐RFA group and 69 in C‐RFA group. There was a significant difference between the two groups (*χ* ^2^ = 4.733, *p* = 0.030). The remaining 137 cases were IDR, including 44 in NT‐RFA group and 93 in C‐RFA group. The two groups did not differ significantly (*χ* ^2^ = 0.425, *p* = 0.514). The 1‐, 2‐, 3‐, 4‐ and 5‐ year LTP‐free survival rate was 87.12%, 79.36%, 74.99%, 72.32%, and 72.32% in NT‐RFA group and 75.75%, 66.52%, 65.52%, 65.52%, and 65.52% in C‐RFA group, respectively. There was a significant difference between the two groups (*χ* ^2^ = 5.061, *p* = 0.024, Figure 2A). The 1‐, 2‐, 3‐, 4‐ and 5‐ year IDR‐free survival rate was 93.04%, 73.02%, 56.08%, 38.16%, and 28.47% in NT‐RFA group and 93.48%, 69.14%, 49.13%, 34.68%, and 28.93% in C‐RFA group, respectively. The two groups did not differ significantly (*χ* ^2^ = 0.185, *p* = 0.667, Figure 2B). Of all cases, only one showed extrahepatic metastasis in C‐RFA group, which was pulmonary metastasis.

### 3.4 Subgroup analysis based on tumor size

**FIGURE 3:**
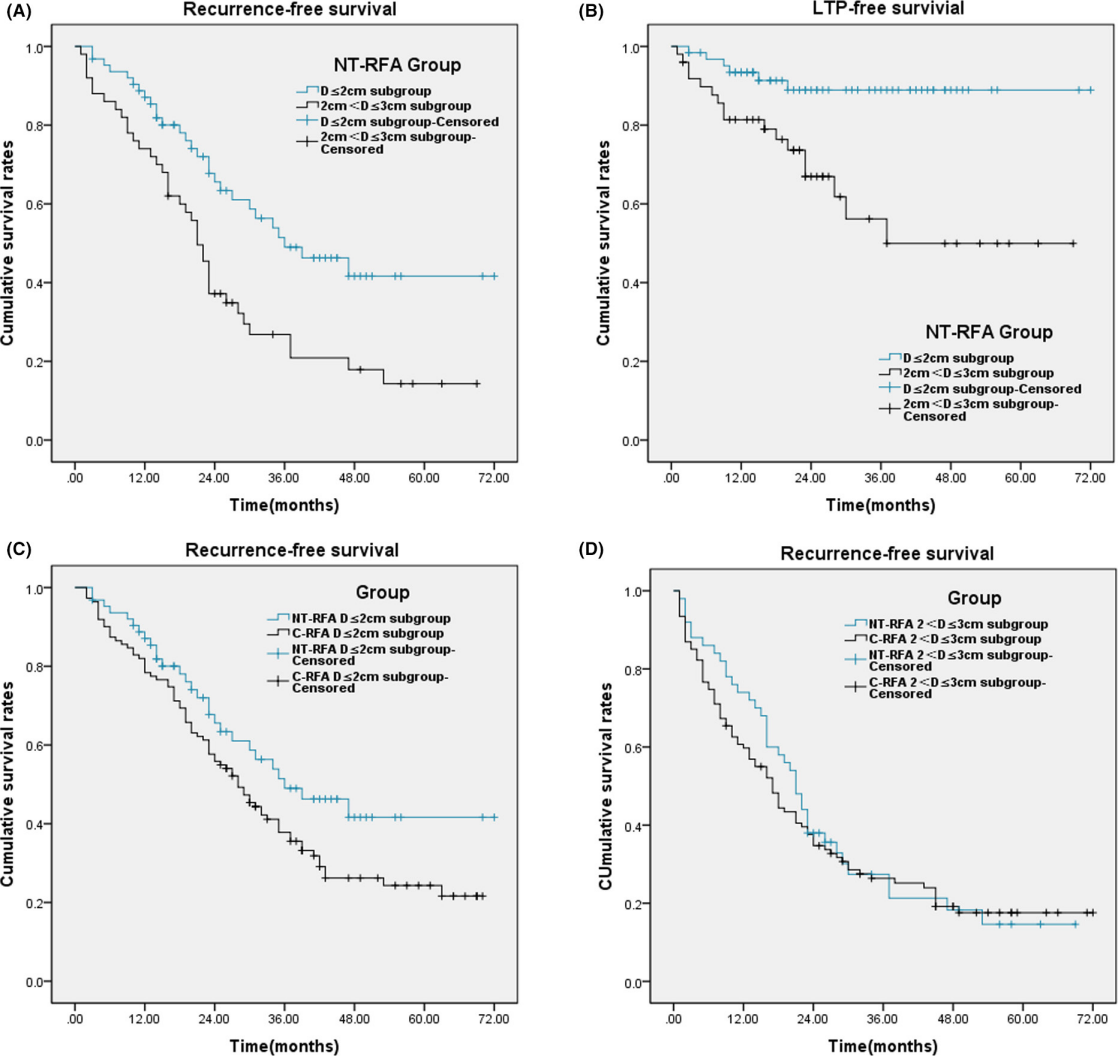
RFS and LTP–free survival in subgroups, according to tumor size. (A) Cumulative RFS rates of D ≤ 2 cm subgroup and 2 cm < D ≤ 3 cm subgroup in NT‐RFA group. There was a significant difference between the two subgroups (*χ* ^2^ = 12.112, *p* = 0.001, log‐rank test). (B) Cumulative LTP–free survival rates of D ≤ 2 cm subgroup and 2 cm < D ≤ 3 cm subgroup in NT‐RFA group. There was a significant difference between the two subgroups (*χ* ^2^ = 12.646, *p* = 0.000, log‐rank test). (C) Cumulative RFS rates of D ≤ 2 cm subgroups in NT‐RFA and C‐RFA groups. There was a significant difference between the two subgroups (*χ* ^2^ = 4.345, *p* = 0.037, log‐rank test). (D) Cumulative RFS rates of 2 cm < D ≤ 3 cm subgroups in NT‐RFA and C‐RFA groups. There was no significant difference between the two subgroups (*χ* ^2^ = 0.309, *p* = 0.578, log‐rank test).

According to the lesion diameter (D), the patients in NT‐RFA group and C‐RFA group were divided into D ≤ 2 cm and 2 cm < D ≤ 3 cm subgroups, respectively. The RFS rate and LTP‐free survival rate were analyzed and compared between the subgroups. In NT‐RFA group, 28 patients (44.44%) showed HCC recurrence in D ≤ 2 cm subgroup (*n* = 63), and 39 (78.00%) in 2 cm < D ≤ 3 cm subgroup (*n* = 50). The 1‐, 3‐ and 5‐year RFS rate was 87.05%, 63.38%, and 41.64% in D ≤ 2 cm subgroup and 76.00%, 34.87%, and 14.31% in 2 cm < D ≤ 3 cm subgroup, respectively. There were significant differences between the two subgroups (*χ* ^2^ = 12.112, *p* = 0.001, Figure 3A). There were six cases (9.52%) of LTP in D ≤ 2 cm subgroup and 17 cases (34.00%) in 2 cm < D ≤ 3 cm subgroup. LTP‐free survival rates differ significantly between the two subgroups (*χ* ^2^ = 12.646, *p* = 0.000, Figure 3B). In C‐RFA group, 78 cases (70.27%) showed HCC recurrence in D ≤ 2 cm subgroup (*n* = 111) and 84 (78.50%) in 2 cm < D ≤ 3 cm subgroup (*n* = 107). There was a significant difference in RFS rate in D ≤ 2 cm subgroups between the two groups (*χ* ^2^ = 4.345, *p* = 0.037, Figure 3C). However, there was no significant difference in RFS rate of 2 cm < D ≤ 3 cm subgroup between the two groups (*χ* ^2^ = 0.309, *p* = 0.578, Figure 3D).

### 3.5 Multivariate analysis of factors affecting HCC recurrence

Multivariate analysis was conducted on seven factors affecting RFS, LTP‐free survival and IDR‐free survival. Five factors significantly affected RFS, including four in NT‐RFA group and four in C‐RFA group. Lesion diameter >2 cm, AFP > 20 ng/mL and safety margin<10 mm were the common significant factors in the two groups. Four factors significantly affected LTP‐free survival, and lesion diameter >2 cm, safety margin<10 mm were risk factors shared by both groups. Four factors significantly affected IDR‐free survival, and only HBV‐DNA > 1000cps/ml was a risk factor shared by both groups. However, the lesion diameter and safety margin were not the risk factors of IDR (Table 3).

**TABLE 3:** Multivariate analysis of the risk factors for RFS, LTP‐free and IDR‐free survival

Variables	NT‐RFA group (*n* = 113)		C‐RFA group (*n* = 218)	
	Hazard ratio (95% CI)	*p* Value	Hazard ratio (95% CI)	*p* Value
Recurrence‐free survival				
Tumor size >2 cm	2.211 (1.278–3.823)	0.005	1.700 (1.224–2.360)	0.002
Cirrhosis (yes)	0.980 (0.512–1.876)	0.951	4.002 (2.229–7.188)	0.000
ALT > 40 U/L	1.195 (0.698–2.044)	0.516	0.897 (0.643–1.252)	0.523
HBV‐DNA(+)	2.210 (1.302–3.750)	0.003	0.845 (0.589–1.213)	0.362
AFP > 20 ng/mL	2.454 (1.405–4.284)	0.002	1.440 (1.040–1.992)	0.028
PIVKA‐II > 90 ng/mL	0.561 (0.292–1.080)	0.084	1.171 (0.829–1.655)	0.370
Safety margin <10 mm	2.436 (1.307–4.542)	0.005	3.589 (1.706–7.547)	0.001
LTP‐free survival				
Tumor size >2 cm	5.351 (1.857–15.418)	0.002	3.785 (2.199–6.516)	0.000
Cirrhosis (yes)	0.965 (0.331–2.813)	0.948	2.955 (1.392–6.271)	0.005
ALT > 40 U/L	1.356 (0.557–3.299)	0.502	1.017 (0.590–1.751)	0.953
HBV‐DNA (+)	0.963 (0.343–2.705)	0.943	0.714 (0.382–1.334)	0.291
AFP > 20 ng/mL	0.758 (0.302–1.903)	0.555	1.800 (1.118–2.900)	0.016
PIVKA‐II > 90 ng/mL	0.754 (0.234–2.429)	0.636	0.809 (0.487–1.344)	0.413
Safety margin <10 mm	6.152 (1.568–24.134)	0.009	4.554 (1.098–18.882)	0.037
IDR‐free survival				
Tumor size >2 cm	1.720 (0.866–3.416)	0.121	1.107 (0.726–1.689)	0.635
Cirrhosis (yes)	1.022 (0.449–2.323)	0.959	4.231 (1.992–8.989)	0.000
ALT > 40 U/L	1.111 (0.566–2.184)	0.759	0.752 (0.491–1.152)	0.190
HBV‐DNA (+)	2.595 (1.346–5.003)	0.004	1.752 (1.142–2.688)	0.010
AFP > 20 ng/mL	4.296 (2.019–9.139)	0.000	1.199 (0.788–1.822)	0.397
PIVKA‐II > 90 ng/mL	0.575 (0.267–1.237)	0.157	1.591(1.017–2.487)	0.042
Safety margin <10 mm	1.851 (0.894–3.832)	0.097	2.022 (0.974–4.198)	0.059

*Note*: Multivariate analyses used Cox proportional hazard model. 95% CI, 95% confidence interval. HBV‐DNA > 1000 cps/ml was considered positive.

## 4 DISCUSSION

In recent years, RFA has been used as the first‐line treatment of early stage HCC, but studies have found that the efficacy of RFA is not as good as surgical resection.^12^, ^13^ Compared with surgical resection, RFA has a higher probability of LTP, resulting in a lower RFS rate.^14^ Conventional RFA has a disadvantage in the treatment of tumor, that is, the electrode is directly inserted into the tumor lesions during the treatment, violating the principle of tumor no‐touch and increasing the probability of tumor diffusion. Moreover, single electrode ablation is prone to incomplete ablation in conventional RFA, leading to residual lesions. Therefore, no‐touch RFA is proposed. In the NT‐RFA procedure, the electrode is avoided from direct contact with the tumor, thus reducing planting and metastasis.^15^ Meanwhile, multi‐electrode ablation can obtain a broader ablation range,^16^ so that the LTP can be reduced and the RFS rate improved.^17^ This technology has been widely used in many HCC treatment centers. However, convincing analysis and study of the long‐term efficacy of NT‐RFA remain to be conducted, and no unified standard for the number of electrodes, operation procedures and scope of application are available. Can NT‐RFA completely replace conventional RFA? There has been no definitive report to date. This is the first study that comprehensively summarizes the experience of applying NT‐RFA to treating patients with single HCC less than 3 cm in diameter in a single center in the past 6 years. All RFA operations were implemented according to unified procedures, excluding technical differences in multicenter studies, with the largest number of cases and the longest duration of follow‐up. In particular, the advantages and disadvantages of NT‐RFA technology were comprehensively analyzed, and the range of HCC patients who can benefit from dual‐electrode NT‐RFA was proposed for the first time.

The main advantage of RFA is minimal invasiveness. While achieving the purpose of lesion destruction, it has the advantages of simple operation, fewer complications, rapid postoperative recovery, short hospital stay and so forth. However, will the use of multi‐electrode in NT‐RFA increase the risk and complications and affect the outcome of patients? Many existing studies have found that multi‐electrode NT‐RFA does not increase complications and is as safe as C‐RFA.^8^, ^18^, ^19^ In this study, dual‐electrode NT‐RFA was used. Compared with C‐RFA, there was no significant difference in operation time, complete ablation rate, complications and hospital stay, but a larger ablation range and safety margin were obtained. Therefore, NT‐RFA is as safe as C‐RFA, and does not increase postoperative complications. Safety margin has been proved to be a key factor that affects survival, especially RFS.^20^ This was also confirmed in this study. Obtaining a larger ablation range and safety margin is one of the main reasons for improvement of LTP by NT‐RFA.

We found that NT‐RFA improved the RFS rate but not OS rate. Therefore, what is the significance of NT‐RFA? Studies have revealed that OS rate of patients with HCC is affected by many factors, mainly liver disease and its complications rather than tumor progression.^21^, ^22^ Most cases in this study had liver disease history, of which 90.94% (301/331) had hepatitis, 82.48% (273/331) liver cirrhosis, 32.33% (107/331) mild to moderate ascites, 20.54% (68/331) portal hypertension, and 2.72% (9/331) a history of upper gastrointestinal bleeding. Meanwhile, 31.72% (105/331) cases were accompanied by hypertension, diabetes, nephropathy, respiratory diseases and other comorbidities. Liver failure, hepatic encephalopathy and upper gastrointestinal bleeding were the main causes of death. For elderly patients, comorbidities are also important factors affecting the OS rate.^20^ In addition, the retreatment methods after HCC recurrence are diversified, including re‐ablation, surgical resection, liver transplantation, transcatheter arterial chemoembolization (TACE), and targeted therapy or immunotherapy. Different retreatment methods have greatly different effects on OS rate.^23^, ^24^, ^25^ Therefore, we assume that RFS rate is more significant than OS rate in evaluating the efficacy of initial treatment. Compared with C‐RFA, NT‐RFA improves the RFS rate, prolongs the time interval of retreatment and reduces the frequency of retreatment.

The causes of LTP include the residual of the original lesion, microvascular invasion and micrometastasis around the original lesion, which usually occurs within 24 months after treatment.^26^ In this study, 95.65% (88/92) of LTP occurred within 24 months after operation. NT‐RFA has been proved to significantly improve the LTP‐free survival rate.^9^, ^10^, ^27^ The reason is that NT‐RFA does not directly contact the tumor and reduces the tumor diffusion and planting metastasis caused by treatment. We speculate that obtaining a larger ablation range and safety margin is the key contributor to LTP decrease after NT‐RFA application. Even for HCC less than 3 cm, there may be microvascular invasion and micrometastasis within 1 cm around the lesion, which is an important factor leading to LTP.^28^, ^29^, ^30^ Therefore, theoretically, the ablation range needs to exceed the lesion boundary by more than 10 mm to obtain a better curative effect.^20^ However, due to the influence of the lesion location and the vessels around the lesion, not all lesions can obtain a safety margin of more than 1 cm. In our study, 42.48% (48/113) of patients had safety margin≥1 cm in NT‐RFA group and 14.22% (31/218) in C‐RFA group, demonstrating significant difference (*χ* ^2^ = 32.705, *p* = 0.000). NT‐RFA with dual‐electrode ablation can more effectively cover the lesion and its surrounding area and reduce the residual lesion. IDR includes intrahepatic metastasis of primary lesions and multicenter growth of HCC,^31^ which mostly occurs 2 years after treatment. At present, most studies have shown that NT‐RFA cannot reduce IDR,^10^, ^32^ which is also confirmed by this study.

In this study, the two groups were divided into D ≤ 2 cm subgroup and 2 cm < D ≤ 3 cm subgroup according to the lesion size. The grouping was based on the operation principle of NT‐RFA. The distance between the two electrode can be adjusted according to the size of the lesion. However, if the distance exceeds a certain range, the ablation of the area between the electrodes will be incomplete, so the distance is limited. Olivier et al reported that the electrode spacing of dual‐electrode NT‐RFA should not exceed 3.0 cm.^33^ In our center, we found that the effective ablation range could not cover all the areas between the electrodes after the electrode spacing exceeded 2.2 cm, which may lead to residual lesions. When treating lesions with a diameter of more than 2 cm, we did not increase the number of electrodes, but used water injection electrode. During the operation, we ensured the water injection direction to face between the electrodes, and slowly injected normal saline during ablation, which can appropriately expand the ablation range.^34^ We found that the efficacy of dual‐electrode NT‐RFA in the treatment of lesions over 2 cm decreased significantly. Compared with C‐RFA, NT‐RFA generated no significant difference in RFS rate and LTP‐free survival rate, suggesting no advantage. Olivier et al. reported that NT‐RFA was used to ablate HCC lesions of 3 cm and above by increasing the number of electrodes (3–4 electrodes) and ablation times, which can also obtain good efficacy.^33^ In contrast, Kayvan et al. believed that the recurrence rate of HCC with a diameter of 2‐5 cm treated with multi‐electrode NT‐RFA was higher than that of surgical resection.^35^ Moreover, increasing the number of electrodes and ablation times will lead to a significant increase in surgical risk and complications,^32^ and the treatment cost will also increase significantly. In addition, RFA is affected by the location of lesions and the structure of hepatic vessels.^36^ NT‐RFA with more than three electrodes is not suitable for all patients and is not conducive to wide use. Despite controversies over the efficacy of multi‐electrode NT‐RFA in the treatment of HCC over 3 cm, we demonstrate that dual‐electrode NT‐RFA can replace C‐RFA in the treatment of small HCC of 2 cm and below.

Introduction of the no‐touch concept into the traditional RFA promotes the progress and development of RFA treatment of HCC, but the single concept of no‐touch is not the key to improving the efficacy. Through multivariate analysis, we believe that the local ablation effect of the lesions is still the key to efficacy. As NT‐RFA improves the local ablation effect, LTP can thus be better controlled. However, due to the limitations of current technology, the efficacy and scope of NT‐RFA application are still affected by the size and location of the lesions, thus preventing the complete replacement of C‐RFA with NT‐RFA. On the other hand, on the basis of the original liver disease, HCC often presents the characteristics of multicenter occurrence and development.^37^, ^38^ Affected by multiple factors, improving local treatment alone is not enough to improve the OS rate of HCC patients, and comprehensive treatment for the occurrence and development of HCC is the key to improving the OS rate.^39^, ^40^, ^41^

In conclusion, compared with C‐RFA, NT‐RFA can better control the LTP of HCC less than 3 cm and significantly improve the postoperative RFS rate. It is a better treatment option for patients with single small HCC. A larger ablation range and safety margin generated by NT‐RFA is the key reason for the improvement of RFS rate, thus significantly reducing LTP. The dual‐electrode NT‐RFA is only suitable for HCC less than 2 cm, in which the RFS rate can be significantly improved, but has no obvious advantage compared with C‐RFA in the treatment of HCC more than 2 cm in diameter.

## AUTHOR CONTRIBUTIONS

Guodong Wu: Methodology, Formal analysis, Writing‐Original Draft Visualization. Jing Li: Investigation, Data Curation. Changfeng Li: Investigation, Data Curation. Xia Ou: Resources, Data Curation. Kai Feng: Resources. Feng Xia: Resources. Zhiyu Chen: Resources. Leida Zhang: Supervision. Kuansheng Ma: Conceptualization, Writing‐Review & Editing, Validation, Project administration.

## FUNDING INFORMATION

This study was supported by the grants from National Natural Science Foundation of China (No. 82073346), and Chongqing Talent Plan Project of Chongqing Health Commission (No. 4246ZP112).

## CONFLICTS OF INTEREST

The authors declared that they have no conflicts of interest to this work.

## GRANTS AND EQUIPMENT

This study was approved by the Research Ethics Committee of the Southwest Hospital, which is the affiliated hospital of the Army Medical University. LDRF‐120 S RF ablation device (Lead Electron Corporation), Elektrotom 106 HiTT minimally invasive surgery system (Berchtold Medizin Electronik GmbH) and EUB‐405 ultrasonic diagnostic instrument (Hitachi)were used.

## Data Availability

Data sharing is not applicable to this article as no new data were created or analyzed in this study.
